# Lane-keeping ability evaluation for driving skill tests: A multi-indicator fusion approach

**DOI:** 10.1371/journal.pone.0329257

**Published:** 2025-08-06

**Authors:** Mengmeng Duan, Hao Wu, Shulin Zhang, Huiqing Jin, Susu Liu

**Affiliations:** 1 Anhui Provincial Key Laboratory of Traffic Information and Safety, Anhui Sanlian University, Hefei, China; 2 School of Intelligent Transportation and Modern Industry, Anhui Sanlian University, Hefei, China; 3 School of Transportation, Southeast University, Nanjing, China; Nanjing Forestry University, CHINA

## Abstract

Traditional driver’s skill tests primarily assess whether candidates meet specific standards in prescribed tasks, which often fails to fully reflect their overall driving performance in real-world scenarios. This can lead to suboptimal driving outcomes. Lane-keeping ability is a key indicator for evaluating a driver’s overall competence, as it reflects their proficiency in vehicle control, road environment perception, and emergency handling. However, due to the complex and varied factors influencing lane-keeping ability, there is currently a lack of effective methods for assessing this skill during drive skill tests. To address this gap, this paper proposes a multi-indicator fusion (MIF) method for evaluating lane-keeping ability in driver skill tests. First, to accommodate real-world lane-keeping scenarios in drive skill tests, multidimensional indicators representing lane-keeping ability are extracted from real low-speed naturalistic driving data, considering both lateral and longitudinal safety and stability. Next, by analyzing the distribution characteristics of these indicators using the K-means clustering method, groups of indicators with similar characteristics are identified. Furthermore, the Youden index, Boxplot, and statistical measures are then employed to determine the threshold values for each indicator, enhancing the accuracy of the evaluation. Finally, a comprehensive evaluation model for lane-keeping ability is constructed using the Analytic Hierarchy Process (AHP) based on a combination of subjective and objective weightings. The proposed MIF-based lane-keeping assessment method for drive skill tests was effectively validated in terms of its rationality and feasibility using naturalistic driving data. This study provides valuable reference points for assessing lane-keeping ability in the context of future autonomous driving environments.

## 1. Introduction

Driver’s skill tests have long been regarded as a fundamental component in ensuring road traffic safety [1,2]. Their effectiveness directly impacts overall traffic safety levels and plays a crucial role in accident prevention. It has been demonstrated that severe traffic accidents have occurred as a result of the driving behaviors of untrained drivers [3–5]. Traditionally, driver skill tests have primarily focused on evaluating a driver’s performance in specific, predefined tasks, assessing whether they meet the established standards. However, this testing approach mainly targets individual skills and often fails to comprehensively reflect a driver’s overall ability and adaptability in real-world driving conditions. As a result, even though a driver may pass the road test, their performance and safety awareness in the complex and dynamic real-world driving environment may still be insufficient, leading to potential traffic safety risks. Therefore, we believe that finding a way to comprehensively assess a driver’s overall driving ability during the road test has become a critical issue that needs urgent attention.

Typically, a driver’s driving process involves stages such as car-following, lane-changing, and free driving. Therefore, most scholars currently focus on driving behavior and driving ability during different stages of the driving process, exploring the factors that influence driver behavior and driving ability. For example, Ge et al. found that a driver’s stress levels and personality significantly affect their driving behavior [6]. Zhang et al. revealed the relationship between different driving styles and vehicle-following characteristics [7], while another study by Zhang et al. used LSTM to explore the interaction between car-following and lane-changing behaviors, highlighting their impact on driving performance [8]. Chen et al. investigated the response of the new follower to a lane-changing maneuver in order to accurately describe vehicle movements in large-scale traffic flow [9]. In addition, safety issues have always been a key focus for many researchers. Shin et al. studied the heterogeneity between vehicle types and driving behaviors, analyzing safe driving behaviors during lane-changing maneuvers [10]. Tan et al. constructed a risk field-based driving behavior model to quantify the safety driving risks during lane-changing and car-following processes [11].

With the development of advanced traffic technologies, recent years have seen a growing focus among scholars on research in the field of intelligent transportation systems (ITS). Several studies have highlighted the impact of advanced technologies on driving behavior and abilities. For example, Wu et al. developed a trajectory control method for CAVs, aiming to enhance the vehicle’s control capabilities in terms of speed and acceleration [12]. Wang et al. applied the Acceleration-Based Collision Criticality Metric for real-time safety capability assessment in autonomous vehicles [13]. Zhang et al. studied safety performance indicators, focusing on speed and acceleration, for Connected and Automated Vehicles (CAVs) at freeway crash hotspots [14]. Chai et al. explored the significance of driver assistance in mitigating distracted driving [15]. Papadoulis et al. examined the impact of autonomous vehicles on highway traffic safety [16]. Additionally, some researchers have investigated methods to enhance driving abilities through advanced technologies. For instance, Robert proposed a data-driven approach to predict driver behavior intentions. Zhao et al. considered the impact of tunnel brightness and noise on drivers and proposed a lane-change model for autonomous vehicles in tunnels based on V2X technology [17]. Schöner et al. introduced a method for safety scoring based on driving style influences, including vehicle distances, time headways, and time to collision, providing potential benefits for the safe operation of autonomous vehicles [18].

Lane-keeping is considered a key factor influencing road traffic safety [19]. Research by Utriainen et al. has also demonstrated the potential safety improvements that lane keeping can bring [20]. As a result, more studies aim to enhance driving safety by improving lane-keeping ability. For example, Chen et al. proposed a lane-keeping control method for autonomous vehicles based on Human-Simulated Intelligent Control (HSIC) to improve the robustness of lane control [21]. Although a few studies focus on the lateral lane-keeping levels of vehicles [22], safety factors in the longitudinal dimension are rarely evaluated, as most studies implicitly treat them as kinematic constraints imposed by the driver to control the distance from the vehicle ahead and avoid collisions. Some studies also evaluate drivers’ driving skills by testing their performance in real-world scenarios. Song et al. assessed and tested key lane-keeping scenarios to validate drivers’ skills [23]. In addition, Zhang et al. proposed an evaluation method in the whole parameter space of a logical scenario [24].

Overall, although research on driving behavior and driving ability has made some progress, most studies have primarily focused on the normal driving behavior of licensed drivers, who are often classified as skilled drivers. However, due to ethical and regulatory issues, data on the driving behavior of unlicensed drivers is scarce, and there is less research on drivers without a license. Currently, driver skill tests are mainly limited to assessing whether a driver can meet basic driving standards in specific tasks. The test content is relatively simple and cannot effectively reflect a driver’s comprehensive driving skills. A driver’s lane-keeping ability is related to their reaction speed, judgment, and vehicle dynamic control. Excellent lane-keeping ability can reflect a driver’s overall driving ability to some extent and has a potential impact on ensuring road traffic safety [25]. However, numerous factors affect a driver’s lane-keeping ability, which requires consideration from multiple dimensions. This helps avoid the limitations and bias in evaluating driving performance. Currently, there is a lack of effective methods to quantitatively assess lane-keeping performance from multiple perspectives, preventing a comprehensive evaluation of a driver’s lane-keeping abilities. To fill this gap, the work considers both lateral and longitudinal dimensions of lane-keeping and proposes a method based on multi-indicator fusion (MIF) to quantify the driver’s lane-keeping performance to assess their lane-keeping ability, providing a reference for driver skill test evaluation, overcoming the limitations of the existing driving skill test program in terms of drivers’ comprehensive ability.

The main contributions of this study are as follows:

(1) In this study, we extracted a dataset that aligns with the lane-keeping characteristics in driving skill tests. We applied the k-means clustering algorithm and spearman correlation coefficient for data analysis. From both the horizontal and vertical dimensions, we identified 10 key metrics to evaluate lane-keeping ability in driving skill tests.
(2) In this study, we determined the optimal thresholds for each indicator by integrating the Youden index, boxplot techniques, and statistical methods.
(3) In this study, we employed the AHP to determine the subjective weights and the entropy weight method to establish the objective weights. The coefficient of variation method was then utilized to derive the combined subjective and objective weights, and developing a comprehensive assessment model for evaluating drivers’ lane-keeping ability.

The rest of this paper is organized as follows: In Section 2, the extraction and processing of the Aerial Dataset for China Congested Highway and Expressway (AD4CHE) are introduced. Section 3 presents a method for determining the evaluation indicators of lane-keeping ability during driver skill tests. In Section 4, we first determined the thresholds and weights of each indicator and then introduced the method for constructing a specific road test lane-keeping ability evaluation model. In Section 5, we discuss the practical value of this work in traditional driver skill tests and future autonomous driving tests. Finally, in Section 6, we draw our conclusions and discuss future research directions.

## 2. Dataset extraction and processing

Due to low-speed driving scenarios being key features in driver skill tests, and it is highly correlated with driving characteristics in congested areas, we use the dataset sourced from the AD4CHE project [26]. By selecting segments from this dataset, we are able to extract samples that meet the research requirements with a higher probability, thus avoiding the issue of evaluation model accuracy caused by sparse data. This dataset was collected via drones and contains traffic data from congested highways and expressways in China. It includes 5.12 hours of aerial footage covering a total distance of 6,540.7 kilometers across four different cities in China, with information on 53,761 vehicles.

**Fig 1:**
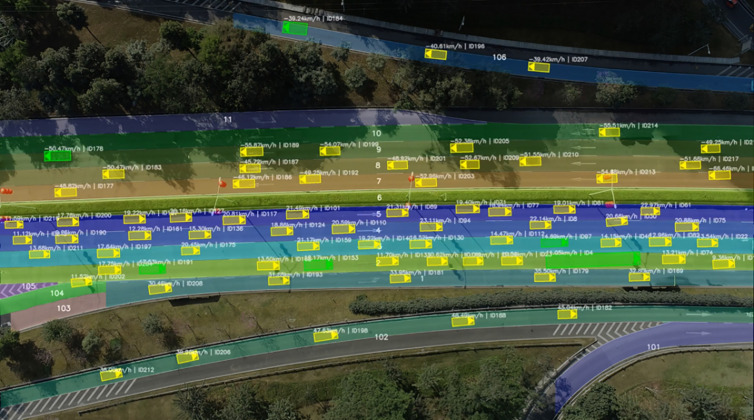
An example of an AD4CHE segment.

The structured data within the dataset includes various parameters such as vehicle position, speed, acceleration, and lane information. Additionally, the dataset provides newly added parameters like lane angle, direction, yaw rate, and vehicle offset, which are valuable for studying subtle behavioral changes in human drivers. Fig 1 shows the actual road environment from one of the aerial data segments. This dataset can be applied to the extraction and analysis of typical driving scenarios, such as lane-keeping, cut-ins, and lane changes. For this work on assessing lane-keeping ability during drive skill tests, we selected the lane-keeping data and processed it to meet the research requirements for evaluating lane-keeping ability.

### 2.1 Data extraction

To ensure that the data meets the requirements for evaluating lane-keeping ability during drive skill tests, we first performed data cleaning to correct any errors in the source dataset. This process included identifying and rectifying anomalies, such as abnormal speed values, acceleration values, and lane offset values. Due to the randomness in the data collected by AD4CHE aerial photography, there may be a few observed sample values that differ significantly from the majority of the samples. Therefore, it is necessary to remove outliers. The study primarily uses the quartile method to eliminate outliers. Specifically, the 25th percentile $Q_{1}$, 50th percentile $Q_{2}$, and 75^th^ percentile $Q_{3}$ of the data are identified, and the interquartile range (IQR) of the data sequence is calculated as equation (1):


$$IQR=Q_{3}-Q_{1}$$ (1)

The study defines data outside the range $\left[Q_{1}-3IQR,Q_{1}+3IQR\right]$ as outliers and removes them.

Next, to align with the characteristics of the road test scenarios in our study, we focused on extracting low-speed lane-keeping scenarios from the dataset. The specific extraction criteria are as follows:

(a) **Lane-Keeping Scenario:** Only segments where the vehicle is driving straight within the lane, without lane changes or line crossings, were selected.
(b) **Low Speed:** Vehicle speed was restricted to less than 30 km/h to facilitate the analysis of low-speed traffic flow characteristics.
(c) **Time Duration:** Segments with a duration of more than 10 seconds were selected to ensure that the segment represents a stable lane-keeping scenario, thereby enhancing the accuracy of subsequent analyses.

**Fig 2:**
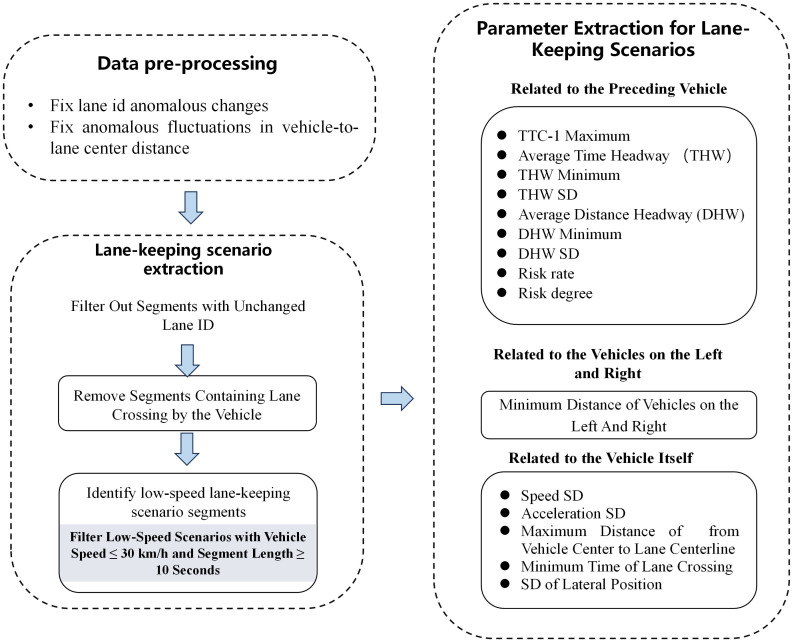
Data extraction flow chart.

The specific extraction process, as shown in Fig 2.

**Fig 3:**
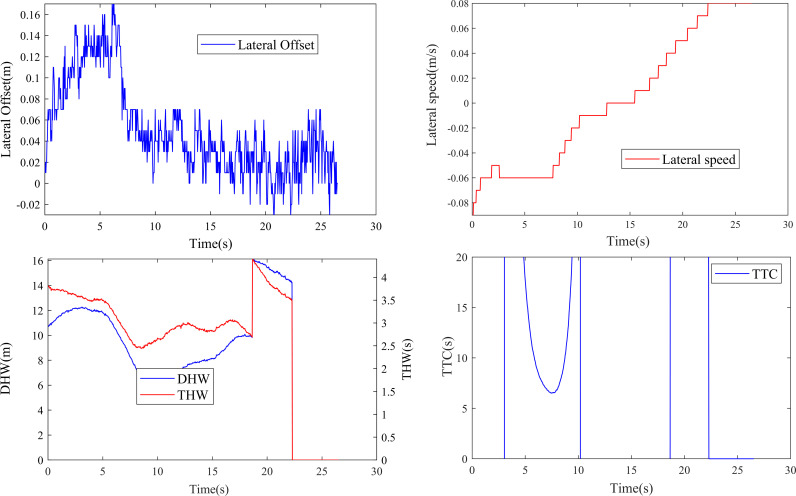
Changes in certain lane-keeping parameters.

After the selection process, a dataset containing 2926 low-speed lane-keeping segments was obtained. Fig 3 presents a better example illustrating the time-series variation of four parameters within one of these lane-keeping segments.

### 2.2 Data processing

To evaluate drivers’ lane-keeping ability during drive skill tests, we identified 15 indicators that can be used for evaluation by reviewing and summarizing key aspects of driver performance in both lateral and longitudinal operations, as shown in Table 1.

**Table 1:** Preliminarily determined lane-keeping ability evaluation indicators.

Dimension	Indicator	Meaning
Longitudinal	TTC^-1^ Maximum (s) [27]	Reciprocal of collision time
	Average THW (s) [28]	Average time headway
	THW Minimum (s) [28]	Minimum time of headway
	THW SD (s) [28]	Time Headway stability
	Average DHW (m) [28]	Average distance headway
	DHW Minimum (m) [28]	Minimum distance of headway
	DHW SD (m) [28]	Distance headway stability
	Speed SD (m/s)	Speed stability
	Acceleration SD (m/s^2^) [29]	Acceleration stability
	Risk rate [30]	Follow-up risk rate
	Risk degree [30]	Follow-up risk degree
lateral	LCO Maximum (m) [31]	Maximum distance from vehicle center to lane centerline
	TLC Minimum (s) [32]	Minimum lane crossing time
	LD Minimum (m) [31]	Minimum distance to the vehicles on the left and right
	SDLP (m) [33]	Standard deviation of lateral position

The aforementioned indicators include TTC, THW, DHW, etc., which describe the relationship between the ego vehicle and the lead vehicle during lane-keeping, as well as indicators such as speed, acceleration, and lateral offset from the lane centerline that describe the state of the ego vehicle.

Some indicators for lane-keeping capability evaluation can be directly provided, such as THW and DHW. However, some indicators require further explanation, such as risk degree, risk rate, and TLC.

The risk rate and risk degree are related to the vehicle’s speed and acceleration. They can be used to describe the risk of collision with the lead vehicle during lane-keeping, which is crucial for maintaining lane safety.

First, the following distance threshold $d(t{)}_{min}$ at time t is calculated based on the speed and acceleration of both the ego vehicle and the lead vehicle at time t. It can be computed using equation (2).


$$d(t{)}_{min}=[v_{e}(t)t_{r}+\frac{1}{2}a(t)t_{r}^{2}+\frac{{\left(v_{e}(t)+t_{r}a(t)\right)}^{2}}{2a_{max,brake}}-\frac{v_{l}{\left(t\right)}^{2}}{2a_{max,brake}}]$$ (2)

where $v_{e}(t)$ and a(t) represent the actual speed and acceleration values of the ego vehicle at time t, respectively; $v_{l}(t)$ represents the actual speed of the ego vehicle; $t_{r}$ represents the reaction time of the ego vehicle.

Next, compare the actual following distance at time t with the minimum following distance $d(t{)}_{\min}$, define the function δ(t), and δ(t)=(0,1), satisfies the function (3).


$$\delta (t)=\{\begin{array}{c} {}*20c1\\ 0 \end{array}\begin{array}{c} {}*20cd(t)\leq d{\left(t\right)}_{\min}\\ else \end{array}$$ (3)

The risk rate $P_{e}$ is represented by the proportion of the time periods with risk relative to the total driving duration, as shown in equation (4).


$$P_{e}=\frac{1}{T}\int_{0}^{T}\delta (t)\tau (t)dt$$ (4)

The risk degree $P_{s}$ is characterized by the sum of the ratios of the difference between the real-time safe distance and the actual safe distance to the real-time safe distance during periods of risk occurrence. This is calculated using equation (5).


$$P_{s}=\frac{\int {\mathrm{\backslash nolimits}}_{0}^{T}\frac{d{\left(t\right)}_{min}-d(t)}{d{\left(t\right)}_{min}}\delta (t)\tau (t)dt}{\int {\mathrm{\backslash nolimits}}_{0}^{T}\delta (t)\tau (t)dt}$$ (5)

In the equation, τ(t) represents the data sampling interval; d(t) represents the actual following distance at time t.

**Fig 4:**
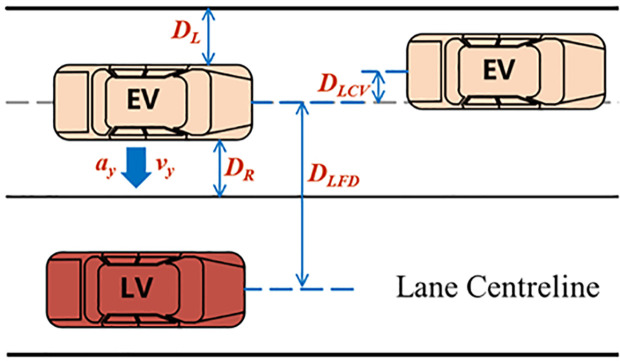
Lane-Keeping Parameter Diagram.


TLC represents the time remaining before the vehicle crosses the lane boundary and can be used to describe the lateral safety margin during lane-keeping. It is one of the key indicators of lane-keeping performance, as shown Fig 4. Equation (6) illustrates the relationship between the time to the left and right boundaries and the vehicle’s state.

$$\{\begin{array}{c} {}*20cv_{y}.TLC+\frac{1}{2}a_{y}.TLC^{2}=D_{L}\;\\ v_{y}.TLC+\frac{1}{2}a_{y}.TLC^{2}=D_{R} \end{array}$$ (6)

In the equation, $v_{y}$ represents the vehicle’s lateral movement speed; $a_{y}$ represents the vehicle’s lateral acceleration; $D_{L}$ represents the distance between the vehicle’s left boundary and the left lane line; $D_{R}$ represents the distance between the vehicle’s right boundary and the right lane line.

**Fig 5:**
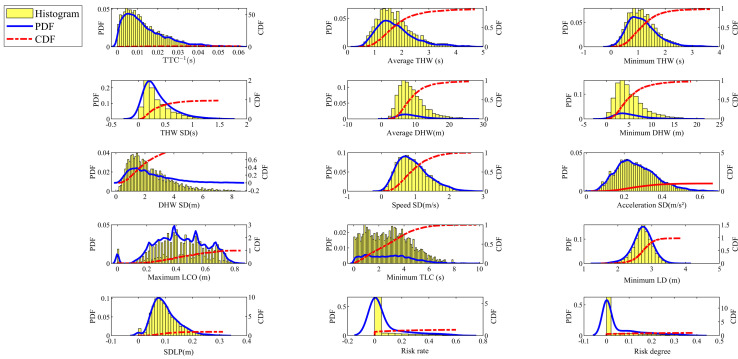
Distribution charts of lane-keeping segments.

To more intuitively represent the distribution of the parameters, the study further presents the data using histograms, as shown in Fig 5.

The distribution histograms of lane-keeping evaluation indicators shown in Fig 5 reveal that most indicators exhibit distribution shapes that deviate slightly from normal distribution, with noticeable skewness and kurtosis. For example, the average THW displays a right-skewed distribution with a kurtosis greater than 3, indicating that a small number of extreme values less than the mean significantly affect the SD. Similarly, the maximum LCO shows a left-skewed distribution, with 20% of drivers managing to keep the deviation within 0.26 m, which is considered a relatively safe range. Additionally, the histograms of risk rate and risk degree exhibit higher bars at 0, followed by a rapid decline, which is mainly due to the majority of scenarios in the dataset being safety-related. These results indicate that the distribution characteristics of the extracted data effectively meet the needs of the research scenario.

To determine the threshold values for lane-keeping evaluation indicators in driving tests, the work applies the K-means clustering method for classification and clustering analysis of various lane-keeping indicators. K-means clustering is an unsupervised learning algorithm with the core idea of assigning data points to K predetermined clusters (where K is the number of clusters specified in advance) and minimizing the squared distance between data points and their respective cluster centers through an iterative optimization process. The ultimate goal of the algorithm is to ensure that each data point is assigned to the cluster center closest to it, thereby achieving optimal classification of lane-keeping data. The specific implementation steps are as follows:

(1) Initialize Centroids: Randomly select K data points as the initial cluster centers.
(2) Assign Data Points: Assign each data point to the cluster corresponding to the nearest centroid.
(3) Update Centroids: For each cluster, calculate the mean of all data points within the cluster and set it as the new centroid.
(4) Iteratively repeat steps (2) and (3) until the centroids no longer change significantly or a predefined number of iterations is reached.
(5) Convergence: After the algorithm converges, each data point is assigned to a cluster, resulting in K clusters.

In the K-means clustering method, selecting an appropriate value for K is a critical issue, as it significantly impacts the determination of threshold values for lane-keeping evaluation indicators in driving tests. In this study, different K values were tested, and the optimal K value was selected through a combination of the silhouette coefficient, the elbow method, and the minimum sample criterion, allowing for the accurate determination of the K value for clustering data.

**Fig 6:**
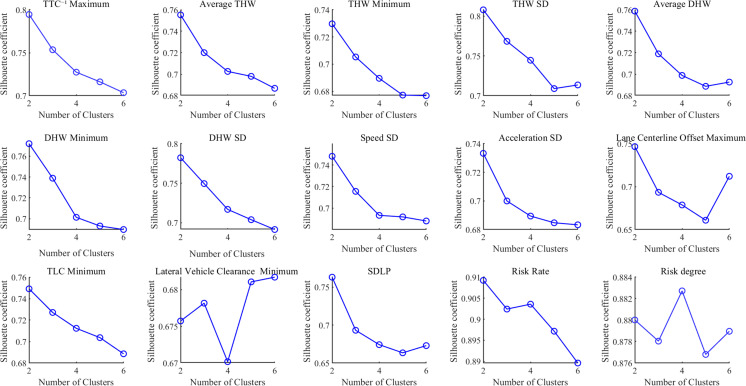
Silhouette coefficients for different clusters of indicators.

First, this study determines the optimal clusters by extracting the silhouette coefficient *S* for different clusters of lane-keeping indicators. The silhouette coefficient S reflects the quality of the clustering, with values ranging from 0 to 1. The closer the value is to 1, the better the clustering performance. The silhouette coefficients S for the indicators across different clusters are shown in Fig 6.

As shown in Fig 6, the silhouette coefficient *S* for the lane-keeping evaluation indicators mostly reaches its maximum value when the number of clusters is set to 2. Additionally, the silhouette coefficients are relatively high across all cluster numbers (ranging from 0.6 to 0.9), indicating good clustering performance.

**Fig 7:**
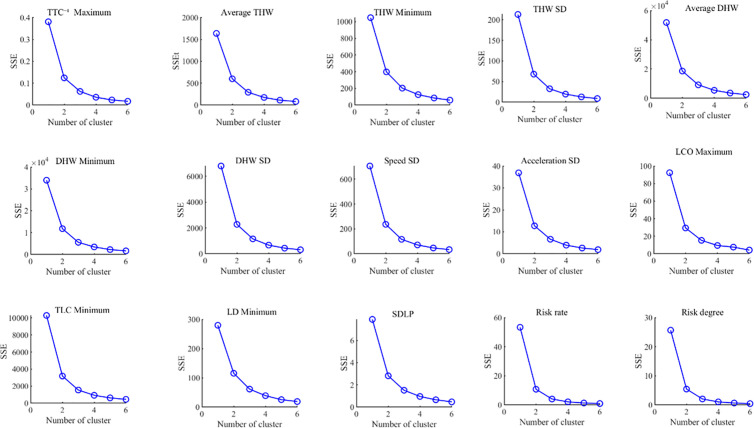
Elbow plot of lane-keeping indicators.

The elbow plot is used to determine the appropriate number of clusters by plotting the trend of the sum of squared errors (SSE) for different numbers of clusters. Typically, as the number of clusters increases, the SSE gradually decreases, but after a certain number of clusters, the rate of decrease slows significantly, forming an “elbow” shape. This point indicates the optimal number of clusters. Fig 7 shows the elbow plot of the sum of squared errors for the lane-keeping evaluation indicators across different numbers of clusters.

As shown in Fig 7, the sum of squared errors for each lane-keeping indicator decreases gradually as the number of clusters increases. The decrease is particularly significant when the number of clusters is 2 or 3, after which the reduction slows down. This indicates that in the elbow plot, the lane-keeping evaluation indicators achieve optimal clustering performance when the number of clusters is 2 or 3.

**Fig 8:**
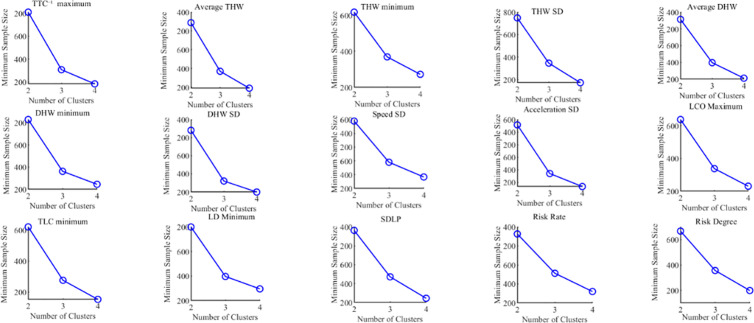
Minimum sample size for different numbers of clusters.

Based on the analysis results of the silhouette coefficient in Fig 6 and the SSE in Fig 7, further analysis was conducted by combining the minimum sample size for each lane-keeping indicator under different numbers of clusters (with the number of clusters being 2, 3, or 4). The results are shown in Fig 8.

As shown in Fig 8, the minimum sample size for each lane-keeping evaluation indicator is largest when the number of clusters is 2, and it decreases progressively, reaching the smallest size when the number of clusters is 4.

**Fig 9:**
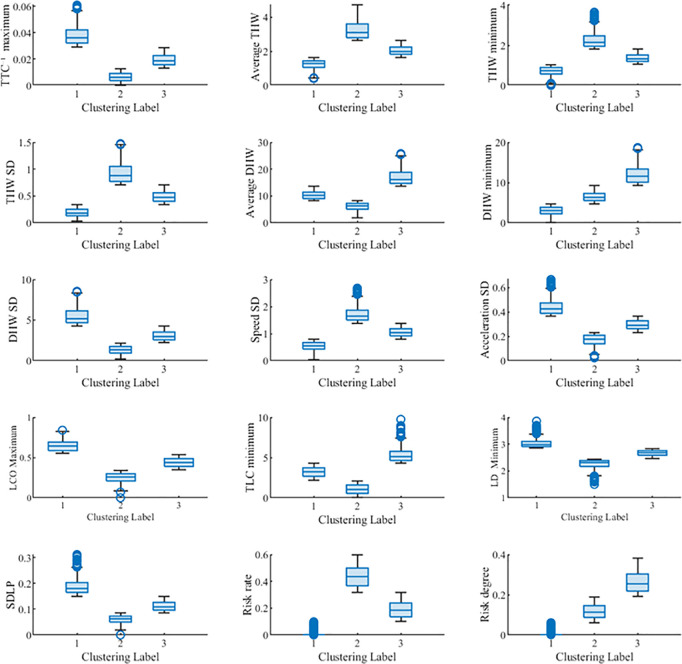
Lane-keeping clustering results for indicators.

Considering the parameter variation characteristics of lane-keeping indicators in Figs 6, 7, and 8, although the clustering performance is better with 2 clusters, limiting the analysis to only 2 clusters may result in a loss of detailed information, leading to an oversimplification of the data. On the other hand, when the number of clusters is set to 3, the clustering performance is only slightly inferior to that of 2 clusters. Moreover, compared to 2 clusters, the 3-cluster division better captures intermediate state samples within the data, more fully reflecting the data’s variability and hierarchical structure. Therefore, the final number of clusters for each evaluation indicator was determined to be 3. The clustering results of the indicators are shown in Fig 9.

From Fig 9, it can be seen that setting the number of clusters for lane-keeping ability indicators to 3 achieves a better clustering result. This is beneficial for determining the thresholds for lane-keeping ability assessment.

## 3. Lane-keeping ability evaluation indicator

In view of the potential issue of feature overlap among the 15 lane-keeping evaluation indicators summarized in the literature. The work employs the Spearman correlation coefficient method to conduct an in-depth analysis of the correlations between the indicators. The aim is to select the highly correlated indicators, thereby avoiding redundancy or repetitive evaluation during the assessment process. The Spearman correlation coefficient method performs well in handling tied ranks and outliers in sequences. We use the correlation coefficient rho(a,b) to represent the correlation between data set a and data set b, as shown in equation (7).


$$rho\left(a,b\right)=1-\frac{6\sum {\mathrm{\backslash nolimits}}_{i=1}^{m}d_{i}^{2}}{m\left(m^{2}-1\right)}$$ (7)

In this equation, $d_{i}$ represents the rank difference between column a and column b; m denotes the length of each column.

**Fig 10:**
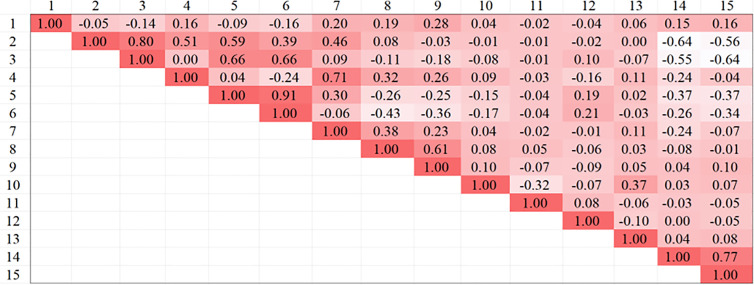
Spearman correlation matrix.

This paper calculated the correlation coefficients among the 15 indicators, resulting in the Spearman correlation matrix shown in Fig 10. To determine the correlation between two parameters, we used a threshold value of 0.7 as the standard [34]. When the correlation coefficient between two parameters exceeds 0.7, it indicates a strong correlation between them.

Fig 10 shows that the correlation coefficient between the THW minimum and the average THW is 0.80, between the DHW SD and the THW SD is 0.71, between the minimum DHW and the average DHW is 0.91, and between the risk rate and risk degree is 0.77. These results indicate a strong correlation between the THW minimum and average THW, the DHW SD and THW SD, the minimum DHW and mean DHW, and the risk rate and risk degree. Consequently, the minimum THW, average DHW, minimum DHW, and risk degree indicators were ultimately excluded. The selected indicators will be used in the subsequent construction of the evaluation model and will provide a reference standard for lane-keeping evaluation in drive skill tests.

## 4. Driver lane-keeping ability evaluation model based on MIF

Through the correlation analysis of low-speed lane-keeping data from AD4CHE, we further identified the indicators that impact a driver’s lane-keeping ability. These indicators are TTC-1, average THW, risk rate, THW SD, DHW SD, speed SD, acceleration SD, maximum LCO, minimum TLC, minimum LD, and SDLP. To comprehensively assess a driver’s lane-keeping ability in drive skill tests, we first determine the threshold values for each indicator. Considering the skewed distribution of the data, this study employs Boxplot, the Youden Index, and the 20%−80% percentiles to determine the thresholds, identify outliers and skewed distributions, and ensure that the threshold setting is not overly affected by extreme data or imbalanced distributions. The study constructs an integrated evaluation model for lane-keeping in drive skill tests based on the AHP and entropy weight method, and it finally proposes a threshold-based method for evaluating a driver’s lane-keeping ability in drive skill tests.

### 4.1 Setting thresholds for evaluation indicators

This work utilizes a combination of the Youden (Y) index, boxplot, and the 20%−80% quantile method to comprehensively determine the optimal thresholds for the evaluation indicators. Relying solely on the Youden index and boxplot to extract outliers for threshold determination may be biased, especially when data distribution is irregular, potentially leading to unreasonable results, such as a negative minimum THW. To ensure the rationality and logical consistency of the data, the optimal thresholds for each indicator were determined by integrating the results from the Youden index and boxplot methods, along with the 20th and 80th percentiles of each indicator. The final optimal thresholds were established by taking the union of these values through logical judgment.

#### 4.1.1 Youden Index.

The Youden Index (Y) [35]is a statistical measure used to evaluate the overall diagnostic performance of a binary classification test or model. It considers both the True Positive Rate (TPR) and the True Negative Rate (TNR), and quantifies the test’s discriminatory ability by providing a single value. The range of the Y index from −1–1, where a value of 1 indicates that the test has perfect discriminatory ability (i.e., both TPR and TNR reach their maximum values). A value of 0 suggests that the test performance is no better than random selection, and a value less than 0 indicates that the test performance is even worse than random selection. Generally, a higher value of the Y index signifies stronger discriminatory ability of the test, with the corresponding threshold being the optimal discrimination threshold [36].The Youden Index can be calculated using Equations (8)-(10).


$$TPR=\frac{TP}{TP+FN}$$ (8)

$$TNR=\frac{TN}{TN+FP}$$ (9)

Y=TPR+TNR−1 (10)

In the formula, TP represents the number of positive samples correctly classified as positive by the model or test; TN represents the number of negative samples correctly classified as negative by the model or test; FP represents the number of negative samples incorrectly classified as positive by the model or test; FN represents the number of positive samples incorrectly classified as negative by the model or test; TPR indicates the proportion of actual positive samples correctly classified as positive by the model or test; TNR indicates the proportion of actual negative samples correctly classified as negative by the model or test.

To calculate the maximum Y value for each indicator in this study, the ROC curve was used to traverse all possible thresholds of the continuous variables based on the clustering results of various parameters extracted from lane-keeping segments. For each threshold, the values of TP, TN, FN, and FP were computed, and the threshold corresponding to the maximum Youden index was determined. The specific discrimination thresholds for the remaining indicators are shown in Table 2.

**Table 2:** Optimal discrimination thresholds for each indicator based on Y index.

Indicator	Discrimination threshold	Indicator	Discrimination threshold
TTC^-1^ Maximum	0.03/s	LCO Maximum	0.44 m
Average THW	1.64 s	TLC Minimum	2.13 s
THW SD	0.70 s	LD Minimum	2.50 m
DHWSD	4.29 m	SDLP	0.15 m
Speed SD	1.38 m/s	Risk rate	0.32
Acceleration SD	0.37 m/s^2^		

By comparing the optimal discrimination thresholds corresponding to the maximum Y values for each indicator with the clustering results, it is evident that the discrimination thresholds for each indicator are very close to the boundary values of the clusters. This indicates that the optimal discrimination thresholds calculated based on Y values possess excellent discriminative ability and are also more precise.

#### 4.1.2 Boxplot.

A boxplot is a visual representation of data, where Q1 is the first quartile, Q3 is the median between the median and the highest value, and the interquartile range (IQR) is defined as Q3 - Q1. Typically, outliers are defined as data points lower than Q1 - 1.5IQR or higher than Q3 + 1.5IQR. Therefore, based on the boxplot, outliers for each indicator are identified, and the values of Q1 - 1.5IQR and Q3 + 1.5IQR for the lane-keeping parameter are used as the passing thresholds. Furthermore, by analyzing how the threshold value of each indicator affects the assessment of the driver’s lane-keeping ability, a single discriminative threshold is ultimately determined. The optimal discrimination thresholds for each evaluation indicator, calculated using the Boxplot method, are shown in Table 3.

**Table 3:** Optimal discrimination thresholds for each indicator based on boxplot.

Indicator	Discrimination threshold	Indicator	Discrimination threshold
TTC-1 Maximum	0.04/s	LCO Maximum	0.94 m
Average THW	0.01 s	TLC Minimum	−2.75 s
THW SD	0.93 s	LD Minimum	2.04 m
DHW SD	−1.13 m	SDLP	0.22 m
Speed SD	2.15 m/s	Risk rate	0.38
Acceleration SD	0.55 m/s^2^		

From Table 3, it can be observed that compared to the thresholds calculated using the Y values, the optimal discrimination thresholds for each indicator obtained through outlier detection exhibit varying degrees of fluctuation. For instance, the thresholds for DHW SD and minimum TLC even resulted in negative values. This is due to the inherent data characteristics of the indicators. For example, the DHW SD indicator, as shown in the frequency distribution histogram in Fig 5, has a wide range between the first quartile and the third quartile, with a relatively small first quartile. This results in negative values when calculated using the boxplot method. Therefore, in future research, this paper will further screen and exclude such data and use multiple cross-validation methods to obtain more scientific and accurate thresholds.

#### 4.1.3 20%−80% percentiles.

By using statistical methods to calculate the mean, median, 20th percentile, 80th percentile, minimum, maximum, standard deviation, kurtosis, and skewness of each lane-keeping evaluation indicator, a more comprehensive understanding of the data’s central tendency, distribution shape, variability, and potential outliers can be achieved. This approach helps in obtaining more accurate thresholds. The statistical values for each indicator are shown in Table 4.

**Table 4:** Statistical values of each indicator.

Indicator	Mean	Median	20th Percentile	80th Percentile	Minimum	Maximum	SD	Kurtosis	Skewness
TTC^-1^ Maximum	0.01	0.01	0.005	0.022	0.00	0.061	0.01	5	1.35
Average THW	1.78	1.64	1.20	2.28	0.41	4.695	0.71	4	1.09
THW SD	0.35	0.27	0.15	0.54	0.03	1.476	0.26	5	1.47
DHWSD	2.30	1.95	1.10	3.36	0.16	8.55	1.45	5	1.26
Speed SD	0.94	0.87	0.47	1.44	0.04	2.68	0.46	3	0.72
Acceleration SD	0.27	0.25	0.18	0.35	0.02	0.67	0.11	3	0.61
LCO Maximum	0.43	0.43	0.26	0.63	0.00	0.84	0.17	3	−0.1
TLC Minimum	2.81	2.73	0.85	4.71	0.00	9.75	1.76	3	0.45
LD Minimum	2.73	2.74	2.51	2.96	1.50	3.86	0.29	4	−0.4
SDLP	0.10	0.09	0.06	0.14	0.00	0.31	0.05	4	0.91
Risk rate	0.07	0.00	0.00	0.13	0.00	0.60	0.13	7	2.15

In summary, both the Y value-based threshold determination and the outlier detection method based on boxplot have certain limitations. Therefore, we further combined the 20th and 80th percentiles of the calculated statistical values for each indicator to determine the optimal thresholds by taking the union of these values based on logical judgment. For example, in the case of the DHW SD, the threshold calculated using the Y value is 4.29m, while the outlier method yields a threshold of 5.79m. The 80th percentile obtained from the statistical distribution is 3.358m. Considering that the dataset mainly consists of licensed drivers with better driving abilities, 5.79m was selected as the threshold for the DHW SD in lane-keeping ability evaluation. Additionally, we excluded clearly unreasonable indicator thresholds. For the TTC^-1^ maximum, we found that the threshold calculated using the Y value is 0.029/s, the outlier detection method gives 0.040/s, and the 80th percentile from the statistical distribution is 0.022/s. It can be observed that TTC^-1^ is significantly lower than the 0.5/s near-collision threshold commonly proposed in most studies [27]. Since the thresholds determined by all three methods are too low, they were deemed unreasonable and excluded. Therefore, the final optimal thresholds for the 10 selected indicators are shown in Table 5.

**Table 5:** Optimal discrimination thresholds for lane-keeping indicators.

Indicator	Discrimination threshold	Indicator	Discrimination threshold
Average THW	1.20 s	LCO Maximum	0.94 m
THW SD	0.93 s	TLC Minimum	0.85 s
DHW SD	5.77 m	LD Minimum	2.72 m
Speed SD	2.15 m/s	SDLP	0.22 m
Acceleration SD	0.55 m/s^2^	Risk rate	0.32

### 4.2 Determination of weights for evaluation indicators

Weights are numerical values used to measure the significance of each indicator within the overall evaluation, describing the importance of individual factors within the factor system. Determining the relative importance of different evaluation indicators allows for a more accurate comprehensive evaluation of a driver’s road test ability. There are many methods for determining indicator weights, generally categorized into subjective weighting methods and objective weighting methods. Subjective weighting is primarily based on the decision-expert’s subjective judgment of the importance of each attribute, with the original data obtained through expert experience and judgment. Objective weighting is primarily determined based on the degree of correlation between indicators or the amount of information provided by each indicator.

To overcome the limitations of using a single weighting method and to fully leverage the strengths of various weighting approaches, this study employs a combination of subjective and objective weighting methods. This combined approach eliminates subjective bias and objective one-sidedness, ensuring that the determined weights reflect both subjective and objective information and accurately and comprehensively represent the actual driving ability assessed in the road test.

#### 4.2.1 Subjective weights based on the AHP.

The AHP, introduced by Thomas L. Saaty of the University of Pittsburgh in 1977 [37], is a method that combines qualitative and quantitative judgments to describe objective evaluations. This study establishes a hierarchical evaluation framework for assessing drivers’ lane-keeping ability in driving skill tests. Based on the characteristics of the evaluation indicators and the final evaluation goals, the problem is developed into a multi-level analysis structure model. This approach scientifically and rationally determines the subjective weights of indicators at each level, enhancing the objectivity and credibility of the evaluation system and providing a more accurate theoretical foundation for subsequent driver training and testing.

The steps for determining the subjective weights of indicators using AHP are as follows.

(1) Construct a hierarchical model

The hierarchical structure model typically includes three levels from top to bottom: the goal hierarchy, the criteria hierarchy, and the indicator hierarchy. The goal hierarchy represents the highest level in the hierarchy, which in this study is the evaluation of drivers’ lane-keeping ability in drive skill tests. The criteria hierarchy consists of the criteria for assessing the quality of the options. In this study, safety and stability are added as judgment criteria, so the criteria hierarchy includes longitudinal safety, longitudinal stability, lateral safety, and lateral stability. The indicator hierarchy consists of the specific influencing factors, which are the 10 determined evaluation indicators. Therefore, this study constructs a hierarchical structure evaluation model for drivers’ lane-keeping ability, divided into four dimensions with a total of 10 indicators, as shown in Table 6.

**Table 6:** Hierarchical structure model for driver lane-keeping ability.

Goal Hierarchy	Criteria Hierarchy	Indicator Hierarchy
Driver Lane-Keeping Ability	Longitudinal Safety	Average THW
		Risk Rate
	Longitudinal Stability	THW SD
		DHW SD
		Speed SD
		Acceleration SD
	Lateral Safety	LCO Maximum
		TLC Minimum
		LD Minimum
	Lateral Stability	SDLP

(2) Constructing the fuzzy judgment matrix

The construction of the fuzzy judgment matrix is a key step in the AHP. This study uses the consistent matrix method proposed by Saaty et al. to construct the judgment matrix, which involves comparing factors pairwise rather than all at once. By using relative scales, this method aims to minimize difficulties in comparing factors with different properties and enhance accuracy, as shown in Table 7.

**Table 7:** The 9-level scale method.

Scale	Meaning
1	Two factors are equally important.
3	One factor is slightly more important than the other
5	One factor is moderately more important than the other
7	One factor is strongly more important than the other
9	One factor is absolutely more important than the other
2,4,6,8	The intermediate value between the two adjacent levels of judgment
Reciprocal	The importance of the comparison when the order of the two elements is reversed

In constructing the judgment matrix, the values for each element are determined by comparing elements pairwise under a certain criterion from the previous level. Therefore, for the criteria hierarchy elements longitudinal safety, longitudinal stability, lateral safety, and lateral stability under the goal hierarchy of assessing drivers’ lane-keeping ability, we can construct a 4x4 judgment matrix A.


$$A=\left[\begin{array}{cccc} {}*20ca_{11} & a_{12} & a_{13} & a_{14}\\ a_{21} & a_{22} & a_{23} & a_{24}\\ a_{31} & a_{32} & a_{33} & a_{34}\\ a_{41} & a_{42} & a_{43} & a_{44} \end{array}\right]$$ (11)

Where, $a_{ij}$ represents the relative importance of element $a_{i}$ with respect to element $a_{j}$ from the perspective of assessing drivers’ lane-keeping ability. That is aij=wi/wiwj\nulldelimiterspacewj. $w_{i}(w_{j})$ represents the weight of the i(j) criterion in the criteria hierarchy with respect to the importance of the goal hierarchy for drivers’ lane-keeping ability. Therefore, the judgment matrix A has the following properties.


$$a_{ii}=1;a_{ij}=\frac{1}{a_{ji}};a_{ij}\geq 0;a_{ij}=\frac{a_{ik}}{a_{jk}}$$

Based on the above principles, the criteria hierarchy judgment matrix is constructed as shown in Table 8.

**Table 8:** Criteria hierarchy judgment matrix.

	Longitudinal Safety	Longitudinal Stability	Lateral Safety	Lateral Stability
Longitudinal Safety	1	5	1	5
Longitudinal Stability	1/5	1	1/5	1/2
Lateral Safety	1	5	1	4
Lateral Stability	1/5	2	1/4	1

Since the judgment matrix should have complete consistency, in practice, however, when using pairwise comparisons, estimation errors may arise due to the limitations of the evaluator’s knowledge and experience. Therefore, a consistency check is further required.

a) Determine the eigenvector of the judgment matrix *A*, which represents the relative weights of each factor.

Normalize each column of the judgment matrix *A*: $b_{ij}=\frac{a_{ij}}{\sum {\mathrm{\backslash nolimits}}_{i=1}^{n}a_{ij}}(j=1,2,\ldots n)$

Sum the rows of the normalized matrix: ${\bar{W}}_{i}=\sum {\mathrm{\backslash nolimits}}_{j=1}^{n}b_{ij}(i=1,2,\ldots n)$

normalized vector $\bar{W}=[\begin{array}{cccc} {}*20c{\bar{W}}_{1} & {\bar{W}}_{2} & \ldots & {\bar{W}}_{n} \end{array}{]}^{T}$: $W_{i}=\frac{{\bar{W}}_{i}}{\sum {\mathrm{\backslash nolimits}}_{i=1}^{n}{\bar{W}}_{i}}$, The elements of *W* represent the ranking weights of the relative importance of the factors from the previous hierarchy with respect to a given criterion in the hierarchy, specifically the ranking weights of the four elements in the criterion hierarchy regarding the lane-keeping ability of drivers in the objective hierarchy. Thus $W=[\begin{array}{cccc} {}*20cW_{1} & W_{2} & \ldots & W_{n} \end{array}{]}^{T}$ is the sought characteristic vector, which is also the result of the pairwise comparison matrix in hierarchical sorting.

Calculate the maximum eigenvalue of the pairwise comparison matrix: $\lambda_{\max}=\frac{1}{n}\sum {\mathrm{\backslash nolimits}}_{i=1}^{n}\frac{{\left(AW\right)}_{i}}{W_{i}}$, $AW_{i}$ is the i component of the vector matrix AW.

The calculated feature vector and weight results for the criterion hierarchy are shown in the Table 9. ($\lambda_{\max}=$ 4.047)

**Table 9:** Feature vector and weight results for the criterion hierarchy.

	Longitudinal Safety	Longitudinal Safety	Longitudinal Safety	Longitudinal Safety
Feature vector	2.236	0.376	2.115	0.562
weight	0.42	0.07	0.40	0.11

(3) Hierarchical ranking and consistency check
  b) consistency check

Since the pairwise comparison matrix is estimated and may contain errors, it is necessary to perform a consistency check. The consistency index (*CI*) is calculated as follows in formula (12).


$$CI=\frac{\lambda \max-n}{n-1}$$ (12)

CI=0 indicates perfect consistency in the pairwise comparison matrix. The larger the CI value, the greater the degree of inconsistency in the matrix. Generally, if CI≤0, the consistency of the matrix is considered acceptable. otherwise, pairwise comparisons should be revised. The larger the dimension of the matrix, the worse the consistency tends to be, so a modification value RI is introduced, as shown in Table 10 [38]. And a more reasonable CR is chosen as an indicator for measuring matrix consistency.

**Table 10:** RI value table (Part).

Number of matrix n	1	2	3	4	5	6	7	8	9
RI	0	0	0.52	0.89	1.12	1.26	1.36	1.41	1.46


$$CR=\frac{CI}{RI}$$ (13)

Thus, if CR ≤ 0.1, the consistency of the pairwise comparison matrix is considered acceptable. otherwise, the pairwise comparisons should be revised [39].

Consistency checks of the weights were performed, and the results are shown in Table 11.

**Table 11:** Consistency check results.

$\lambda_{\max}$	CI	RI	CR	Consistency check results
4.047	0.016	0.89	0.018	CR < 0.1, pass

The results show that the maximum eigenvalue $\lambda_{\max}$ is 4.047. According to the Table 11, the corresponding RI is 0.89. Thus, $CR=\frac{CI}{RI}=0.018$ < 0.1, the consistency check is passed, it is demonstrated that the weight determination method is reasonable, and there is no need to modify the judgment matrix.

(4) Overall ranking and consistency check

In order to determine the final weights of each element in the hierarchical model, it is necessary to perform a comprehensive ranking, which involves calculating the relative importance of all factors with respect to the highest level (the goal level). This process is carried out from top to bottom, progressing sequentially from the highest level to the lowest, the specific representation is as follows.

Assume A is the goal level, including n factors $B_{1},B_{2},...,B_{n}$, The weight coefficients for their hierarchical single ranking relative to $A_{j}$ are $b_{1j},b_{2j},....b_{nj}(j=1,2,,....m)$, Then the overall importance of the B hierarchy in the total ranking is given by $b_{i}=\sum {\mathrm{\backslash nolimits}}_{j=1}^{m}a_{j}b_{ij}(i=1,2,...,n)$. That is, the overall importance of criterion hierarchy $B_{i}$ is the weighted sum of the relative importance of the elements in the upper hierarchy $A_{j}$. Its specific representation is shown in Table 12.

**Table 12:** Overall importance of hierarchy B.

Hierarchy A Hierarchy B	$\begin{array}{cccc} {}*20cA_{1} & A_{2} & \cdots & A_{m} \end{array}$	Overall ranking of hierarchy B
$\begin{array}{cccc} {}*20ca_{1} & a_{2} & \cdots & a_{m} \end{array}$
*B*_*1*_	$\begin{array}{cccc} {}*20cb_{11} & b_{12} & \cdots & b_{1m} \end{array}$	$b_{1}=\sum {\mathrm{\backslash nolimits}}_{j=1}^{m}a_{j}b_{1j}$
*B*_*2*_	$\begin{array}{cccc} {}*20cb_{21} & b_{22} & \cdots & b_{2m} \end{array}$	$b_{2}=\sum {\mathrm{\backslash nolimits}}_{j=1}^{m}a_{j}b_{2j}$
…	…	…
*B*_*n*_	$\begin{array}{cccc} {}*20cb_{n1} & b_{n2} & \cdots & b_{nm} \end{array}$	$b_{n}=\sum {\mathrm{\backslash nolimits}}_{j=1}^{m}a_{j}b_{nj}$

The overall importance (total ranking) must also undergo consistency checking. The method involves evaluating from high to low. For example, the consistency indicator for the relative importance of certain factors in hierarchy B with respect to $A_{j}$ is denoted as $CI_{j}$, and the modification value is $RI_{j}$. The random consistency ratio for the overall importance of hierarchy B is denoted asCR, it can be calculated by equation (14). When CR≤1, the hierarchical overall importance is considered satisfactory. Otherwise, the values in the pairwise comparison matrix need to be adjusted.


$$CR=\frac{\sum {\mathrm{\backslash nolimits}}_{j=1}^{m}a_{j}CI_{j}}{\sum {\mathrm{\backslash nolimits}}_{j=1}^{m}a_{j}RI_{j}}$$ (14)

We ultimately obtain the comprehensive weights of lane-keeping ability evaluation indicators based on hierarchical analysis, as shown in Table 13.

**Table 13:** Subjective weights of each indicator determined based on AHP.

Goal hierarchy	Criteria hierarchy	weight	Indicator hierarchy	Weight	Comprehensive weight
Driver Lane-Keeping Ability	Longitudinal Safety	0.4	Average THW	0.35	0.14
			Risk rate	0.65	0.26
	Longitudinal Stability	0.1	THW SD	0.61	0.06
			DHW SD	0.09	0.01
			Speed SD	0.15	0.02
			Acceleration SD	0.15	0.02
	Lateral Safety	0.4	LCO Maximum	0.14	0.06
			TLC Minimum	0.53	0.21
			LD Minimum	0.33	0.13
	Lateral Stability	0.1	SDLP	1	0.10

#### 4.2.2 Objective weight determination based on the entropy weight method.

The entropy weight method is an objective weighting method that determines objective weights based on the amount of information contained in the indicators. If the entropy of an evaluation indicator is low, it indicates that the variability of the indicator value is high, meaning it provides more information and plays a more significant role in the evaluation of drivers’ lane-keeping ability during drive skill tests. Consequently, its weight should be higher. Conversely, if the entropy of an evaluation indicator is high, it suggests that the variability of the indicator value is low, indicating that it provides less information and plays a less critical role in the evaluation of lane-keeping ability, and thus, its weight should be lower. Therefore, this paper determines the objective weights of each evaluation indicator for lane-keeping based on information entropy, providing a basis for the comprehensive evaluation of drivers’ lane-keeping ability through multi-indicator fusion.

We assume there are n segments of drivers’ lane-keeping data and m evaluation indicators, which together form the original data evaluation matrix X.


$$X=\left[\begin{array}{cccc} {}*20cx_{11} & x_{12} & \cdots & x_{1m}\\ x_{21} & x_{22} & \cdots & x_{2m}\\ \vdots & \vdots & \ddots & \vdots \\ x_{n1} & x_{n2} & \cdots & x_{nm} \end{array}\right]$$ (15)

Therefore, the steps for determining the objective weights of multiple indicators based on the entropy weight method are as follows:

(1) Data Normalization

Firstly, the 10 evaluation indicators identified in this work are classified into positive and negative indicators. Positive indicators are those where higher values indicate better characteristics, including the average THW, minimum TLC, and minimum LD. Negative indicators are those where lower values indicate better characteristics, including risk rate, THW SD, DHW SD, speed SD, acceleration SD, maximum LCO, and SDLP. The specific classification of positive and negative indicators, along with some statistical values, is shown in the Table 14.

**Table 14:** Statistical values of each evaluation indicator.

Indicator	Mean	Median	Minimum	Maximum	SD	positive (+)/negative (-)
THW	1.78	1.64	0.41	4.695	0.71	+
THW	0.35	0.27	0.03	1.476	0.26	–
DHW	2.30	1.95	0.16	8.55	1.45	–
Speed	0.94	0.87	0.04	2.68	0.46	–
Acceleration	0.27	0.25	0.02	0.67	0.11	–
LCO Maximum	0.43	0.43	0.00	0.84	0.17	–
TLC Minimum	2.81	2.73	0.00	9.75	1.76	+
LD Minimum	2.73	2.74	1.50	3.86	0.29	+
SDLP	0.10	0.09	0.00	0.31	0.05	–
Risk rate	0.07	0.00	0.00	0.60	0.13	–

From the Table 14, it can be observed that the scales of the evaluation indicators differ, and some indicators (such as THW and Risk rate) have significantly different maximum values, which greatly affect the weight distribution. Therefore, it is necessary to first standardize the data of each indicator, converting it to the range [0,1].

Let the standardized matrix be denoted as Z, Let the elements in Z be denoted as $Z_{ij}$。

Positive Indicator: $z_{ij}=\frac{x_{ij}-\min\{x_{1j},x_{2j},\ldots ,x_{nj}\}}{\max\{x_{1j},x_{2j},\ldots ,x_{nj}\}-\min\{x_{1j},x_{2j},\ldots ,x_{nj}\}}$

Negative Indicator: $z_{ij}=\frac{\max\{x_{1j},x_{2j},\ldots ,x_{nj}\}-x_{ij}}{\max\{x_{1j},x_{2j},\ldots ,x_{nj}\}-\min\{x_{1j},x_{2j},\ldots ,x_{nj}\}}$

In the formula, min $\left\{x_{1j},x_{2j},\ldots ,x_{nj}\right\}$ is the minimum value of the evaluation indicator $X_{j}$, max$\left\{x_{1j},x_{2j},\ldots ,x_{nj}\right\}$ is the minimum value of the evaluation indicator $X_{j}$, They can be found in Table 13.

(2)The information entropy $E_{j}$ represents the amount of information contained in the indicator, which indicates the importance of the attribute. It can be calculated using formula (16).


$$E_{j}=-\frac{\sum {\mathrm{\backslash nolimits}}_{i=1}^{n}p_{ij}\log(p_{ij})}{\log(n)}$$ (16)

In the formula, $p_{ij}=\frac{z_{ij}}{\sum {\mathrm{\backslash nolimits}}_{i=1}^{n}z_{ij}}$ represents the proportion of the current element in the current feature column. In general, $E_{j}\geq 0$. If $p_{ij}=0$, then $E_{j}=0$. The information entropy for each evaluation indicator is calculated as shown in Table 15.

**Table 15:** Entropy of each evaluation indicator.

indicator	*X*_*1*_	*X*_*2*_	*X*_*3*_	*X*_*4*_	*X*_*5*_	*X*_*6*_	*X*_*7*_	*X*_*8*_	*X*_*9*_	*X*_*10*_
*E*	0.980	0.994	0.996	0.996	0.994	0.995	0.990	0.975	0.996	0.996

where $X_{i}$ refers to the indicators corresponding to Table 2.

Therefore, the weights $W_{j}$ of each indicator can be calculated using formula (17) as shown in the Table below. Specific values can be found in the Table 16.

**Table 16:** Objective weights of each evaluation indicator.

Indicator	*X*_*1*_	*X*_*2*_	*X*_*3*_	*X*_*4*_	*X*_*5*_	*X*_*6*_	*X*_*7*_	*X*_*8*_	*X*_*9*_	*X*_*10*_
*W*	0.23	0.07	0.04	0.04	0.07	0.05	0.12	0.29	0.04	0.04


$$W_{j}=\frac{1-E_{j}}{m-\sum {\mathrm{\backslash nolimits}}_{j=1}^{m}E_{j}}$$ (17)

From Table 16, it can be observed that the highest objective weight is assigned to *X*_*8*_, which represents the minimum TLC for lateral safety, with a weight of 0.29. This is followed by the average THW, representing longitudinal safety, with a weight of 0.23. The weights of the THW SD, DHW SD, the minimum LD, and SDLP are the smallest, each being 0.04. Additionally, a comparison with the subjective weights reveals that both the objective and subjective weights for the minimum TLC are relatively high, while the weights for the DHW SD are the lowest in both objective and subjective evaluations.

#### 4.2.3 Subjective and objective combined weights based on AHP-entropy.

The linear weighting method is used to combine the determined subjective and objective weights. Specifically, the combined weights for the indicators of drivers’ lane-keeping ability during drive skill tests are calculated using the coefficient of variation [40]. For the weights derived from both the entropy weight and the AHP, as shown in formula (18). This approach effectively overcomes the limitations of relying on a single method, making the final weights for the evaluation indicators more scientifically reasonable. It provides a more accurate, objective, and comprehensive reflection of drivers’ lane-keeping ability.


$$W_{i}^{\prime }=\lambda \alpha_{i}+(1-\lambda )\beta_{i}$$ (18)

In the formula, $\alpha_{i}$ represents the subjective weights obtained from the AHP; $\beta_{i}$ represents the objective weights obtained from the entropy weight method; $W_{i}^{\prime }$ denotes the combined weights; λ is the proportion of subjective weights in the combined weights, which can be calculated using formula (19) based on the coefficient of variation method.


$$\lambda =\frac{n}{n-1}[\frac{2}{n}(P_{1}+2P_{2}+\cdots +nP_{n})-\frac{n+1}{n}]$$ (19)

In the formula, $P_{i}(i=1,2,\ldots ,n)$ represents the vector of subjective weights sorted in ascending order, n is the number of evaluation indicators. The final weight coefficients determined based on AHP-Entropy are shown in Table 17.

**Table 17:** Results of subjective and objective combined weighting.

	Indicator	Subjective weight	Objective weight	Combined weight	
Longitudinal Safety	Average THW	0.14	0.23	0.19	0.36
	Risk rate	0.26	0.07	0.17	
Longitudinal Stability	THW SD	0.06	0.04	0.05	0.14
	DHW SD	0.01	0.04	0.02	
	Speed SD	0.02	0.07	0.04	
	Acceleration SD	0.02	0.05	0.03	
Lateral Safety	LCO Maximum	0.06	0.12	0.09	0.43
	TLC Minimum	0.21	0.29	0.25	
	LD Minimum	0.13	0.04	0.09	
Lateral Stability	SDLP	0.10	0.04	0.07	0.07

From Table 17, it is evident that both the subjective and objective weights of the average THW, representing longitudinal safety, and the TLC minimum, representing lateral safety, are relatively high. This underscores their significance in evaluating lane-keeping ability. Although the objective weight of the risk rate is comparatively lower, it is rated highest in terms of subjective importance. Therefore, the use of combined weights results in a more comprehensive and balanced evaluation, mitigating the potential bias of relying on a single weight. Moreover, the consistent weighting across longitudinal and lateral dimensions suggests that these factors are equally important in the context of driver skill tests. Additionally, the higher weights assigned to safety indicators over stability indicators highlight the critical role of safety in the lane-keeping process.

### 4.3 Threshold-based grading evaluation model for drivers lane-keeping ability

Based on the determined thresholds for each indicator and the combined weights, a threshold-based evaluation model for drivers’ lane-keeping ability during drive skill tests is constructed.


$$S=100\;\sum {\mathrm{\backslash nolimits}}_{i=1}^{10}W_{i}\;Z_{i}$$ (20)

In the formula, S represents the total quantified score of drivers’ lane-keeping ability during drive skill tests; $W_{j}$ is the combined subjective and objective weight for each indicator; $Z_{i}$ is the value of each indicator after normalization to the [0,1] range using the thresholds applied to natural driving data; $W_{i}\cdot Z_{i}$ represents the passing score for each indicator based on the thresholds. The passing scores for each indicator and the total quantified threshold score of the model are calculated according to the above formula and are shown in Table 18.

**Table 18:** Full scores and passing scores for various lane-keeping indicators.

Indicator	Combined weight $W_{i}$	Full Score	Threshold	Normalized threshold *Z*_*i*_	Passing Score *S*
Average THW	0.19	19	1.204	0.135	2.56
Risk rate	0.17	17	0.320	0.582	9.89
THW SD	0.05	5	0.927	0.381	1.91
DHW SD	0.02	2	5.790	0.322	0.64
Speed SD	0.04	4	2.147	0.135	0.54
Acceleration SD	0.03	3	0.546	0.190	0.57
LCO Maximum	0.09	9	0.935	0.001	0.009
TLC Minimum	0.25	25	0.847	0.088	2.20
LD Minimum	0.09	9	2.715	0.516	4.64
SDLP	0.07	7	0.217	0.325	2.28
Sum	1.00	100			**25.24**

**Fig 11:**
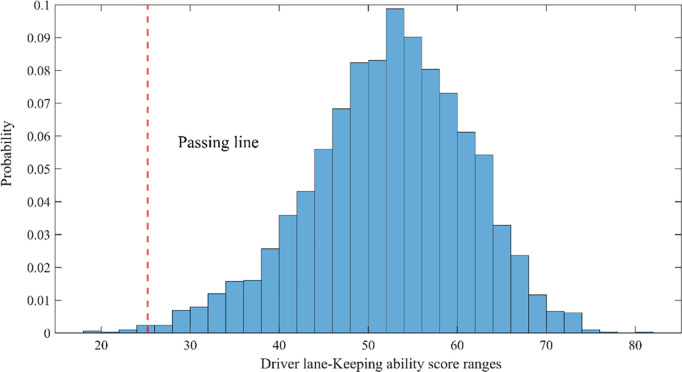
Comprehensive evaluation results of drivers’ lane-keeping ability based on natural driving data.

From the results in Table 18, it can be seen that if the comprehensive score of each indicator in the drivers’ lane-keeping ability evaluation is greater than 25.24, it is considered that the driver has passed the lane-keeping ability test. The evaluation of drivers’ lane-keeping ability in the natural driving dataset is shown in Fig 11.

As shown in the Fig 11, the distribution of driver scores follows a normal distribution. Among them, 99.56% of drivers fall within the passing range, with the highest score being 80.1. Out of 2,926 driving segments, only 13 drivers’ lane-keeping abilities were rated as failing.

## 5. Conclusion

Due to the current driver skill tests primarily assessing whether drivers meet specific standards in individual tasks, they are limited in evaluating only a single skill and cannot comprehensively reflect the driver’s overall competence. This study considers the importance of lane-keeping ability in assessing a driver’s overall driving skills, investigating a lane-keeping ability evaluation method based on MIF for driver skill tests, aiming to provide a reference for enhancing the current driver skill testing framework. Specifically, the work first analyzes natural driving data of human driving behavior in typical low-speed lane-keeping scenarios. Ten indicators used for assessing driver lane-keeping ability were extracted from both lateral and longitudinal dimensions. Next, a combination weighting model of subjective and objective weights using AHP and entropy weight method was employed to determine the weights of each indicator. The Youden Index, Boxplot, and statistical values of the indicators were analyzed to determine the thresholds for each indicator. Based on the determined weights and thresholds, a comprehensive evaluation model was constructed. Finally, the model was applied to actual natural driving data for evaluating driver lane-keeping ability. The evaluation results validate the accuracy and effectiveness of the proposed method for assessing driver lane-keeping ability. This work provides an effective comprehensive evaluation method for driver skill testing, enhancing the effectiveness of driver skill training. Moreover, it can also contribute to the testing of lane-keeping assistance system (LKAS) functionalities in high-level autonomous vehicles, thereby improving the safety and reliability of LKAS technology.

This study also has several limitations. The validation of our MIF-based lane-keeping ability evaluation method is primarily based on existing natural driving datasets. During actual testing, there is a possibility of long-tail events in which a driver may pass the lane-keeping ability evaluation despite exhibiting an abnormal value in a specific indicator. The scalability and adaptability of this method in complex real-world environments still need to be further improved. With the gradual advancement of autonomous driving technology and the increasing maturity of driver assistance systems, driving skill test vehicles will extensively utilize advanced information technologies. In the future, we will focus on the real-time data collection capabilities of high-level autonomous vehicles, exploring real-time lane-keeping ability assessment methods for driver skill test, it will provide scientific guidance for evaluating the safety and reliability of autonomous vehicles in real-road operations, and ultimately help explore methods to ensure road traffic safety from the source.
